# Recombinase Polymerase Amplification Based Multiplex Lateral Flow Dipstick for Fast Identification of Duck Ingredient in Adulterated Beef

**DOI:** 10.3390/ani10101765

**Published:** 2020-09-29

**Authors:** Ming Fu, Quanwang Zhang, Xiang Zhou, Bang Liu

**Affiliations:** 1 Key Laboratory of Agricultural Animal Genetics, Breeding, and Reproduction of Ministry of Education & Key Laboratory of Swine Genetics and Breeding of Ministry of Agriculture, Huazhong Agricultural University, Wuhan 430070, China; fuming@webmail.hzau.edu.cn (M.F.); quanwangzhang@webmail.hzau.edu.cn (Q.Z.); 2 The Cooperative Innovation Center for Sustainable Pig Production, Huazhong Agricultural University, Wuhan 430070, China

**Keywords:** meat adulteration, beef, duck, recombinase polymerase amplification, multiplex lateral flow dipstick

## Abstract

**Simple Summary:**

The adulteration and authenticity of meat and meat products has become a global social problem. Beef is often intentionally adulterated with cheap meat. In order to ensure the authenticity of meat, and provide technical support to regulatory authorities, we developed a rapid and visual method to detect duck ingredient in adulterated beef. This method is implemented recombinase polymerase amplification (RPA) and multiplex lateral flow dipstick (MLFD) cascade. The whole RPA-MLFD reaction process can be finished within 35 min, and the results can be determined by naked eyes. RPA-MLFD was applied to simultaneously detect duck ingredient and beef ingredient without using additional instruments. An adulteration ratio as low as 5% of duck ingredient in beef can be easily measured. Moreover, we confirmed that our new method held good potential in the detection of commercially processed meat samples. Therefore, this study reports a useful animal derived meat adulteration detection method, which have potential application in future.

**Abstract:**

Meat adulteration has become a global social problem. In order to protect consumers from meat adulteration, several methods have been developed to identify meat species. However, the conventional methods are labor-intensive, time-consuming and require instruments. In the present study, a rapid and visual method based on recombinase polymerase amplification (RPA) and multiplex lateral flow dipstick (MLFD) was developed to detect duck ingredient in adulterated beef. Using recombinase and strand displacement polymerase enable RPA to amplify different double-labeled DNA amplicons at room temperature, which can be further detected by MLFD. The whole reaction process can be finished within 35 min, and the results can be determined by naked eyes. As low as 5% of duck ingredient in adulterated beef can be easily measured. Moreover, we confirmed that our new method held good potential in the detection of commercially processed meat samples. In conclusion, this study reported a useful animal derived meat adulteration detection method, which have potential application in future.

## 1. Introduction

Recently, the adulteration and authenticity of meat and meat products has become a global social problem. One of the main kind of adulterations in meat and meat products is the partial or total replacement of high valued meat by cheap ones for economic benefit [1]. Beef is one of the most widely eaten meats in the world; the global beef consumption was close to 60 million tons in 2017 according to data from China Business Industry Research Institute [2]. However, beef is most frequently adulterated mainly due to its high price and wide consumed [3,4]. The European horse meat scandal in 2013 was a famous example of beef adulteration, where horse meat was disguised and sold as beef, which raised social concerns about the authenticity of meat and processed meat products [5]. Many beef products and related products are reported to be adulterated with cheap meats, such as pork, chicken, and duck. Moreover, duck is one of the most commonly used materials to mix or replace beef because of the similar meat color and texture and the lower price [6,7]. Presently, numerous news media have reported on the emergence of various beef products being adulterated with duck in the Chinese market [8,9]. Therefore, the development of fast and accurate identification methods is essential to ensure the food safety and authenticity of meat and meat products.

Various methods including protein- and DNA-based assays have been developed to detect meat authenticity [10,11,12,13,14]. However, the identification of meat species mainly adopts DNA-based nucleic acid detection methods, of which the most widely used methods are the polymerase chain reaction (PCR) based methods [15,16]. In addition, nucleic acid isothermal amplification techniques have been developed rapidly in recent years. Among isothermal amplification techniques, recombinase polymerase amplification (RPA) only requires a pair of primers to complete the amplification of a particular DNA fragment [17]. In order to replace opening target DNA strand by thermal denaturation, the RPA reaction operates isothermally by using enzymes to separate the double-stranded DNA [18]. The exponential amplification can be detected within 30 min in a temperature range of 37 to 42 °C [19]. Recently, several applications of RPA in DNA and RNA target detection have been proved [20,21], and the integration of RPA in different platforms has been reported [22,23,24]. Lateral flow dipsticks (LFD) has great application prospects in the detection field due to easy-to-use, high sensitivity, less time consuming and low-cost [25,26,27]. Furthermore, RPA products can be detected with lateral flow dipstick (LFD) which only requires 5 min to complete all test process without any special equipment, and the results can be observed by naked eyes [28]. For these reasons, the combination of isothermal RPA and lateral flow dipstick can be used as a rapid detection method to identify animal components in adulterated meat.

In this study, a feasible assay combining RPA with multiplex lateral flow dipstick (MLFD) for detection of duck ingredients in adulterated beef was developed. Firstly, the RPA primer and probe sets were optimized to produce a large amount of double-stranded DNA products labeled with biomarkers for beef and duck ingredient detection, respectively. Then, the multiplex lateral flow dipstick was well-integrated for the rapid and easy visible detection of duck ingredient in adulterated beef within 35 min. The developed method is relatively easy to operate and has high sensitivity. Moreover, the method could be really applied to rapid on site test for meat authenticity in future.

## 2. Materials and Methods

### 2.1. Sample Preparation and DNA Extraction

Raw meat samples (beef, mutton, duck, pork and chicken) and commercially processed beef samples (beef dumplings, beef ham and beef slice, *n* = 2 of each sample) were purchased from local supermarkets (Wuhan, China), and the raw meat samples were taken from skeletal muscle of each specie. All samples were cut into small pieces using a sterile scalpel and stored at −20 °C for further use. The adulterated beef samples were treated based on the standard protocols. In general, fresh meat was cut into small pieces and dried at 65 °C. Then, the meats were ground to powder separately, and the adulterated beef samples were prepared by mixing with duck in a series of proportions of 5%, 25%, 50% and 100% (*w*/*w*), respectively. The genomic DNA was extracted using proteinase K-SDS-phenol/chloroform protocol [29]. DNA concentration and purity were measured with NanoDrop 2000 Spectrophotometer (Thermo Fisher Scientific, Waltham, MA, USA). The DNA extracts were stored at −20 °C until further analysis.

### 2.2. RPA Primer and Probe Design

DNA sequences were obtained from Ensembl database (http://asia.ensembl.org/index.html). The candidate sequences were tested for homology with other species using online Basic Local Alignment Algorithm Search Tool (BLAST). The genomic sequences for Bovine (Gene number: ARS-UCD1.2:23:10955159:10956296:1) and Anatinae (Gene number: ENSAPLG00000007071) were selected as target sequences. RPA primers and probes were designed by Primer 5.0 software. All primers and probes were synthesized by Shanghai Generay Biotech Co.Ltd. (Shanghai, China). One primer pair and probe for each specie were selected to do further test (Table 1). The 5′ end of reverse primer for beef identification was labeled with biotin, and the 5′ end of reverse primers for duck detection was labeled with digoxin. The 5′ end of the probes was labeled with fluorescein isothiocyanate (FITC). The C3 blocker were attached to the 3′ end of the probe, which could prevent nucleobase from polymerizing during amplification. In addition, the insertion of an abasic furan (dSpacer) which was introduced in the probe sequence to mimic an abasic site.

### 2.3. Preparation of AuNPs and AuNP-FITC Conjugates

Gold nanoparticles (AuNPs) were prepared by sodium citrate reduction method [30]. A total of 200 mL of ultrapure water was continuously heated and rotated to boiling, then 2 mL of chloroauric acid (HAuCl_4_·3H_2_O, final concentration = 0.01%) and 3.6 mL of 1% trisodium citrate were added until the solution became wine-red. Considering the important role of protein and pH in the binding of AuNPs to protein complexes, the pH and protein content were optimized. After the AuNPs were cooled to room temperature, 15 μL of 0.01 M K_2_CO_3_ and 6 uL of monoclonal FITC antibody were added to 1 mL of AuNPs solution to adjust the pH and protein. Subsequently, 1.13 mL of 10% BSA solution and 292 uL of 2% PEG20000 were added and mixed to block the excess reaction sites of AuNPs, 10% sucrose and 0.01% Tween-20 were added to increase the surface activity of AuNPs. Finally, the AuNPs were centrifuged at 4 °C and 2000× *g* for 30 min, and then the supernatant was removed and the obtained particles were suspended in 1 mL of borax buffer as AuNP-FITC conjugates.

### 2.4. Preparation of Lateral Flow Dipsticks

**Figure 1:**
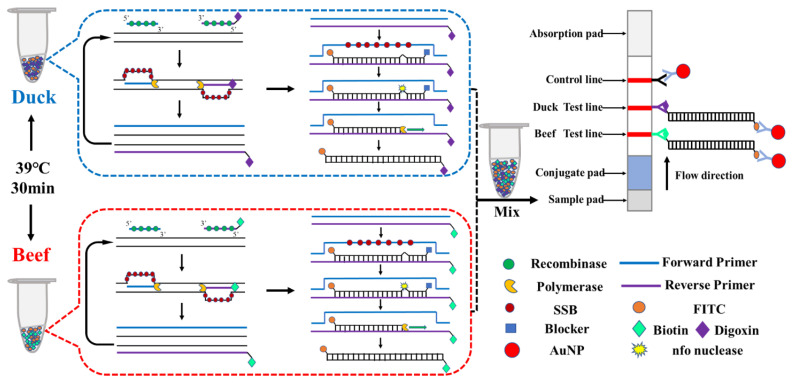
Recombinase polymerase amplification (RPA)–multiplex lateral flow dipstick (MLFD) assay. Firstly, the two oligonucleotide primers form a complex with the recombinase proteins. This complex is able invade the target DNA and directs the primer to homologous sequences. A continuous amplification at 39 °C takes place by strand-displacement synthesis catalyzed by a DNA polymerase, while single-strand binding proteins (SSB) stabilize the displaced strand. Then biotin-labeled or digoxin-labeled nucleic acid amplification products could be hybridized with fluorescein isothiocyanate (FITC)-labeled specific probes. The nuclease nfo contained in the reaction solution recognized the abasic site and cleaved the phosphodiester bond to create a free hydroxyl end where DNA polymerase could extend and continue the amplification process. Finally, the dual-labelled amplicon for beef (biotin-RPA product-FITC) and duck (digoxin-RPA product-FITC) were generated. The mixed RPA amplification were dipped into lateral flow dipstick for MLFD detection.

The LFD was assembled with four parts on a plastic backed sheet in the PVC card, including a sample pad, a conjugate pad, a nitrocellulose membrane, and an absorbent pad (Figure 1). Firstly, the AuNP-FITC conjugates were evenly coated on the conjugate pad of the dipsticks, then placed in an oven at 60 °C for 15 min to 20 min for drying. Then, monoclonal Biotin antibody and Digoxin antibody were diluted to 1 mg/mL in PBS solution and spotted on a nitrocellulose membrane, generating the beef and duck test line (T line), respectively. The FITC secondary antibody was diluted to 1 mg/mL in PBS solution and spotted on a nitrocellulose membrane to generate the control line (C line). Finally, the nitrocellulose membrane was dried at 37 °C for 30 min.

### 2.5. RPA for DNA Amplification

RPA amplification was carried out with the TwistAmp nfo kit (TwistDx, Cambridge, UK). Each reaction was performed in a 23.7 μL reaction mixture containing 15 μL of rehydration buffer, 0.8 μL of forward and reverse primers (10 μM each), 0.3 μL of RPA probe (10 μM), 4.8 μL ddH_2_O and 2 μL of the DNA template. Then, the reaction mixture was added to the freeze-dried tube in TwistAmp nfo kit. To initiate the reaction, 1.3 μL of 280 mM magnesium acetate (MgAc) was added. The reaction temperature and time were 39 °C and 30 min through the optimization. The obtained RPA products were electrophoresed on a 2% agarose gel stained with GelRed (Biotium, California, CA, USA) in 1 × TAE buffer.

### 2.6. Detection of Processed Meat Samples with RPA-MLFD

For processed meat samples detection, RPA amplifications were carried out with the beef and duck specific primers and probes. Then, the two amplification products were mixed in one tube, and 5 μL of the mixed amplification products were diluted in 55 μL of PBS solution. The dilution was put on the multiplex lateral flow dipstick for 2 to 5 min to observe the results.

## 3. Results and Discussion

### 3.1. Design of RPA-MLFD Assay

In order to fast determine duck ingredient in adulterated beef, the recombinase polymerase amplification combined with multiplex lateral flow dipstick (RPA-MLFD) was developed. The first part of RPA-MLFD is to perform RPA reaction with genomic DNA extracted from beef and duck, respectively. After a thermostatic water bath at 39 °C, RPA reaction could amplify target genomic DNA and generate double labelled detectable amplificons (Figure 1).

The visual interpretation of the RPA products was performed on multiplex lateral flow dipstick, and the principle was illustrated (Figure 1). The RPA products for beef and duck, as well as running buffer were mixed in one reaction tube, then the MLFD was dipped into the mixture. The sample solutions could migrate through the whole stripe, and passed the conjugate pad. Then, the FITC-labeled amplification products were captured by anti-FITC-AuNPs conjugate and generating antigen-antibody-AuNPs complex. The immune complex diffused through the chromatographic membrane until to the test (T) lines, and were captured on T lines. The beef T line contains biotin ligand and captured immune complex derived from beef sample, while the duck T line contains digoxin ligand and captured immune complex derived from duck meat sample. Meanwhile, excess anti-FITC-AuNPs conjugate that were not captured T lines continue to diffuse to the control line (C line) and were captured by the secondary antibody to form a color band (Figure 1).

### 3.2. Specificity and Optimization of RPA-MLFD Assay

**Figure 2:**
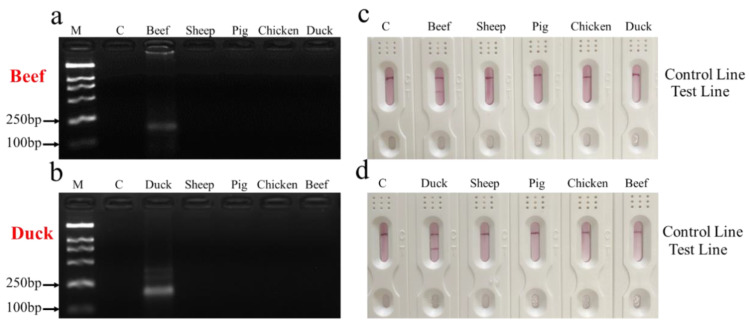
Specificity analysis of beef (**a**,**b**) duck by RPA with agarose gel electrophoresis. Lane M: DL2000 Marker, Lane C: blank; specificity analysis of beef (**c**,**d**) duck by RPA with lateral flow biosensor. Lane C: blank.

**Figure 3:**
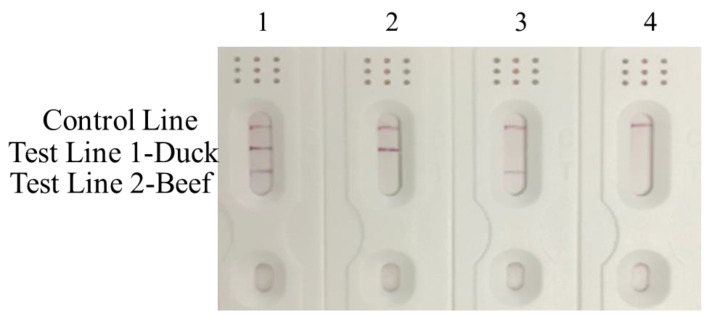
Performance of the RPA-MLFD assay. Lane 1: beef + duck; Lane 2: duck; Lane 3: beef; Lane 4: negative control.

The specificity of primers and probes play critical role in the species identification of meat and meat products. To evaluate the specificity of RPA-MLFD, the genomic DNA extracted from beef and duck was tested by RPA-MLFD. Consistent with the expected results, only the positive sample have amplified products for gel electrophoresis detection. The sizes of the RPA products specific to beef and duck were 234 and 226 bp, respectively (Figure 2a,b). For dipstick detection, only the T line appeared in the successful amplification using beef and duck DNA as template, respectively. No amplification was observed for the negative DNA or blank (Figure 2c,d). In order to improve the performance of RPA-MLFD, the most suitable conditions for the RPA-MLFD assay were explored with sets of different temperatures and times for each species. The optimal reaction temperature and reaction time were 39 °C and 30 min (Figure S1). In addition, when amplification products were carried out with MLFD test, the visualized results for different amplification products were consistent with expectations (Figure 3). Compared to PCR-based methods, the whole procedure of RPA-MLFD for meat specie identification is more rapid (<35 min), easy to operate as it could be carried out at a low temperature (37–42 °C) without professional equipment, and RPA results can be directly visualized on the dipsticks [28,31,32,33]. Moreover, the design of RPA amplification is relatively simple, only one pair of primers and one probe are needed to complete amplification, which is more convenient than isothermal LAMP [34,35]. These advantages fully showed that the established method was well adapted for detection of meat adulteration.

### 3.3. Sensitivity of RPA-MLFD Assay

**Figure 4:**
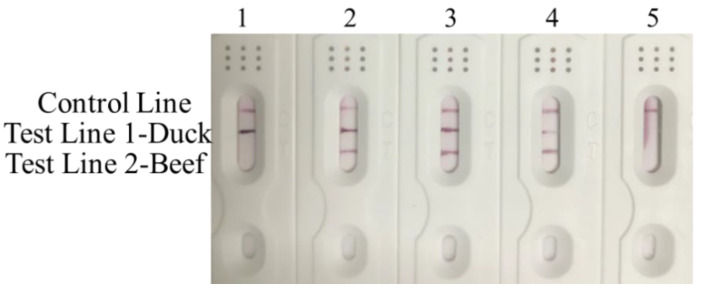
Sensitivity analysis of RPA-MLFD assay for adulterated meat. Lane 1:100% duck; Lane 2: 50% duck + 50% beef; Lane 3: 25% duck + 75% beef; Lane 4: 5% duck + 95% beef; Lane 5: negative control.

To assess the sensitivity of RPA-MLFD assay in adulterated beef samples detection, raw meat mixtures containing a various concentration of 5, 25 and 50% duck in beef were performed to test. The results had shown that all samples could be detected by two T lines and found that the 5% duck in beef can be easily detected (Figure 4). We were not pursuing a lower proportion because it was difficult to obtain any commercial profit from the 5% adulteration of low-quality meat in actual manufacturing and sales, which was in good agreement with the data reported in many previous reports [35,36]. In addition, the sensitivity test was also conducted for each species, a dilution series of beef and duck DNA with concentrations ranging from 100 to 0.01 ng were performed. The method was found to be highly sensitive with a detection limit of 0.1ng for beef and duck (Figure S2a,b), which was equivalent to conventional PCR [37,38]. All the results showed that the RPA-MLFD assay had a great sensitivity without any instrument.

### 3.4. Application of RPA-MLFD in Processed Meat Samples

**Figure 5:**
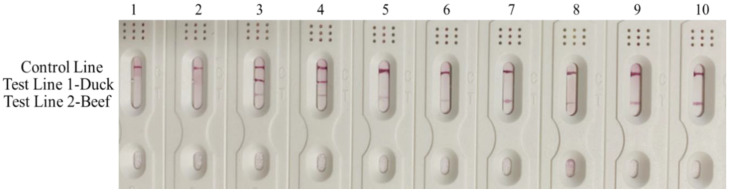
Detection results of real commercial processed meat samples. Lane 1–2: Blank; Lane 3–4: 50% duck + 50% beef adulterated artificial sample; Lane 5–6: beef dumplings; Lane 7–8: beef ham; Lane 9–10: beef slices.

Finally, in order to confirm the practicability of the established RPA-MLFD method, real commercially processed beef products were detected by RPA-MLFD. The results in Figure 5 reveal that all of the commercial products can be accurately detected with our developed method, demonstrating our method was suitable for the detection of processed meat samples. In addition, changing the functional primer and probe sets in RPA-MLFD assay could easily expand the detection strategy to identify other adulterated ingredients accurately and quickly. Furthermore, the RPA-MLFD method requires lesser hands-on time and is easy to perform, the low demand for instruments would make the established method achieve expected on-site detection in future. Therefore, the RPA-MLFD method has a broad application prospect in the authenticity detection of animal derived meat.

## 4. Conclusions

In this study, a novel assay based on isothermal RPA and multiplex lateral flow dipstick detection was demonstrated. The assay was applied to detect duck ingredient in adulterated beef. In this assay, RPA used recombinase and strand displacement polymerase to amplify a specific target sequence at a constant reaction temperature. Using a special probe structure and nuclease, a double-labeled DNA amplicon was generated in one reaction, which could be detected on same one dipstick. The detection limit of RPA-MLFD method is 0.1ng for beef and duck, and an adulteration ratio as low as 5% of duck in beef can be easily measured. Comparison with conventional PCR methods, another advantage of RPA-MLFD method is the constant low temperature required for amplification and the fast reaction speed from the start of the reaction to reading of results in less than 35 min. In addition, the analysis of the reaction itself and lateral flow dipstick don’t require any or only minimal equipment, which is ideal for field testing. An easy-to-see band on one lateral flow dipstick gave a clear yes/no answer, visible to the naked eye and untrained personnel, which was particularly important in remote areas where there may not be trained workers. Therefore, the RPA-MLFD method could be really applied to the meat authenticity detection on site in future.

## Figures and Tables

**Table 1:** The sequence information of primers and probes.

Species	Primer	Sequence (5′–3′)
Bovine	BF	CAGACAAAGGTCAGGAAGTAATCCCAGCGCT
	BR	Biotin–ATTCCTCCAGCCCCCCAGCCGTATTCC
	BP	FITC–CTTGCCCCAAGATGTGGCCTCCAGTTCCCTG dSpacer(THF)–CAAGACTGTAGCCC–C3 Spacer
Anatinae	AF	CCCCAAAGTGTCAACGATTGCCCCGAAACC
	AR	Digoxin–ACGCCCTCATCTCCAAAATCTACCCCAGCC
	AP	FITC–GCCGTCAAAGTCCCCCAAAACACCCTGAAAC dSpacer(THF)–CCCCCAAACCACCGA–C3 Spacer

## Supplementary Materials

The following are available online at https://www.mdpi.com/2076-2615/10/10/1765/s1, Figure S1. Reaction temperature and time optimization of beef and duck primers and probes. Figure S2. Sensitivity of RPA-MLFD for beef and duck primers and probes.
