# Supplementary material for: Recombinase Polymerase Amplification Based Multiplex Lateral Flow Dipstick for Fast Identification of Duck Ingredient in Adulterated Beef
Source: Animals (Basel). 2020 Sep 29;10(10):1765. doi: 10.3390/ani10101765 (PMC7601885; doi:10.3390/ani10101765)
Supplement: Supplementary file 1 [file animals-10-01765-s001.zip › Supplementary Figures/Supplementary Fig.1.pdf]

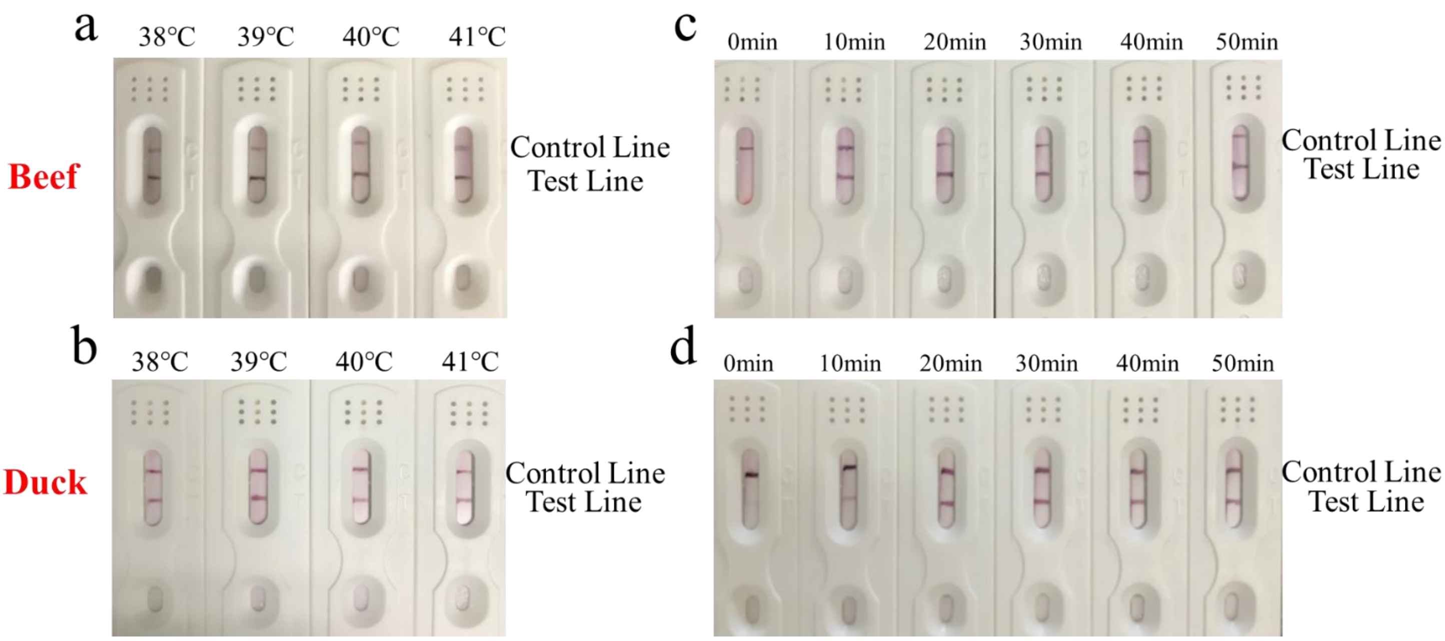

Figure S1. Reaction temperature and time optimization of beef (a, c) and duck(b, d) primers and probes: the reaction temperature range was 38-41°C for each specie; the reaction time was setted as 0min, 10min, 20min, 30min, 40min and 50min for each specie .
