# Supplementary material for: Recombinase Polymerase Amplification Based Multiplex Lateral Flow Dipstick for Fast Identification of Duck Ingredient in Adulterated Beef
Source: Animals (Basel). 2020 Sep 29;10(10):1765. doi: 10.3390/ani10101765 (PMC7601885; doi:10.3390/ani10101765)
Supplement: Supplementary file 1 [file animals-10-01765-s001.zip › Supplementary Figures/Supplementary Fig.2.pdf]

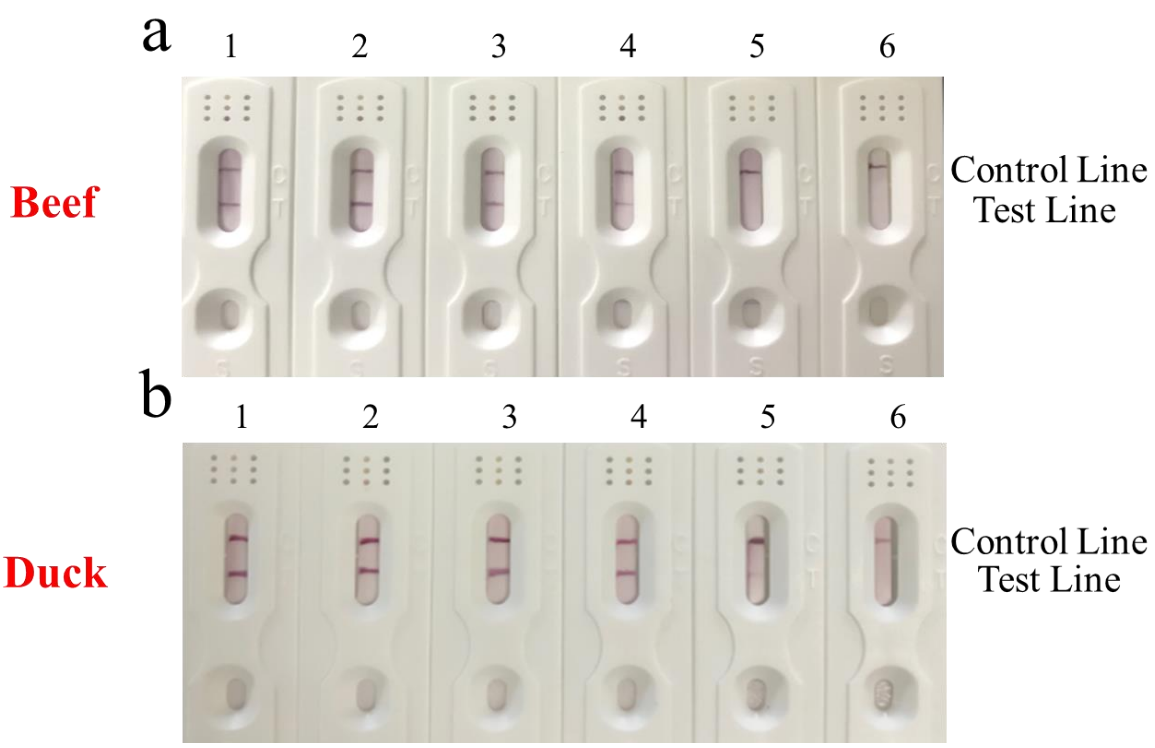

Figure S2. Sensitivity of RPA-MLFB for beef (a) and duck (b) primers and probes; (a) Lane 1-6 : beed DNA quality ranging from 100 ng to 0.01 ng for ten times dilution; (b) duck DNA quality ranging from 100 ng to 0.01 ng for ten times dilution.
